# Indocyanine green used in association with a surgical hemostatic agent as a fiducial marker to reduce overflow during robot-assisted thoracic surgery

**DOI:** 10.36416/1806-3756/e20250090

**Published:** 2025-07-22

**Authors:** Guilherm e Falleiros Mendes, Marcelo Froeder Arcuri, Priscila Mina Falsarella, Alberto Rassi, Rodrigo Gobbo Garcia

**Affiliations:** 1. Centro de Medicina Intervencionista, Hospital Israelita Albert Einstein, São Paulo (SP) Brasil.

## TO THE EDITOR:

Unsuccessful localization of pulmonary nodules during video-assisted thoracic surgery is the most common reason for conversion to thoracotomy. Fiducial markers such as hook wires, microcoils, and liquid markers such as methylene blue have been used in order to assist in locating subcentimeter pulmonary nodules during video-assisted thoracic surgery.^1^ More recently, robot-assisted thoracic surgery (RATS) has been used as a minimally invasive surgical technique for pulmonary nodule resection. However, the presence of robotic arms over the patient may prevent appropriate C-arm positioning and the use of radiopaque markers. 

Indocyanine green (ICG) is a near-infrared fluorescent dye that is used as a liquid fiducial marker with great sensitivity and specificity for direct visualization in the near-infrared spectrum of light.^2^ ICG is advantageous over metal markers such as coils, with reports of fewer complications; does not require intraprocedural imaging; and does not dictate the surgical approach.^3^ Liquid fiducial markers such as methylene blue, India ink, and radionuclides are comparable to ICG but have limitations such as radiation exposure and lower sensitivity.^1^ However, overflow, which is the migration of a liquid fiducial marker beyond the target lesion, has been reported to be more common with ICG than metallic marker migration.^2^ This study sought to describe a fiducial marker technique in which ICG is used in association with a surgical hemostatic agent with tissue adhesive properties to reduce overflow. 

This was a single-center retrospective study approved by the local institutional review board. A search was conducted in the interventional radiology database of a tertiary care medical center to identify patients who underwent preoperative percutaneous localization of lung lesions between May of 2021 and December of 2023. A total of 18 patients (25 lesions) were included. 

Lesions were marked with a 40-slice multidetector CT scanner (SOMATOM Definition AS; Siemens Healthineers, Erlangen, Germany) under general anesthesia. Patients were then sent to an operating room for resection or to a hybrid operating room with a robotic C-arm (Artis zeego; Siemens Healthineers) for cone-beam CT. All localization procedures were performed by an experienced interventional radiologist assisted by a fellow interventional radiologist. All surgical procedures were performed by the local thoracic surgery team, which is experienced in RATS. 

A coaxial introducer needle was percutaneously inserted into the target lesion under fluoroscopic guidance. Further cone-beam CT images or CT images were acquired to confirm the position of the needle. A 5 mg/ml solution of ICG was prepared in a 10 ml syringe, with only one drop being placed within one of the chambers (the thrombin chamber) of the double-chamber (2 ml + 2 ml) syringe for the hemostatic agent TISSEEL (Baxter International Inc., Deerfield, IL, USA), thus creating a solution with a concentration of approximately 0.125 mg/ml of ICG. The double-chamber syringe was then attached to the coaxial needle, and up to 0.5 mL was injected through the mandrel. The inner stylet was used in order to push any remaining solution inside the coaxial needle lumen. The system was then removed. RATS was performed, and pulmonary nodules were located by using a near-infrared fluorescence thoracoscope (da Vinci Firefly; Intuitive Surgical, Inc., Sunnyvale, CA, USA). A wedge resection of the nodule was then performed, and accuracy was confirmed by pathological examination. 

A total of 18 patients underwent preoperative percutaneous localization of lung lesions between May of 2021 and December of 2023. Of the 18 patients, 12 (66.7%) were women and 6 (33.3%) were men. The mean age of the patients was 66 ± 14.06 years. A total of 25 lesions were analyzed. Of the 18 patients who underwent preoperative percutaneous localization of lung lesions during the study period, 13 (72.2%) underwent localization of one lesion, 3 (16.7%) underwent localization of two lesions, and 2 (11.1%) underwent localization of three lesions. 

Pathological examination of the lung lesions showed that 14 of the 18 patients had malignant disease (primary lung cancer, in 12; renal cancer metastasis, in 1; and colon cancer metastasis, in 1) and 4 had benign disease (granuloma, in 2; fibrosis, in 1; and lymphoid hyperplasia, in 1). Mean lesion size was 1.2 ± 0.96 cm (range, 0.3-4.4 cm). Mean lesion-pleura distance was 1.48 ± 1.47 cm (range, 0.1-5 cm). 

**Figure 1:**
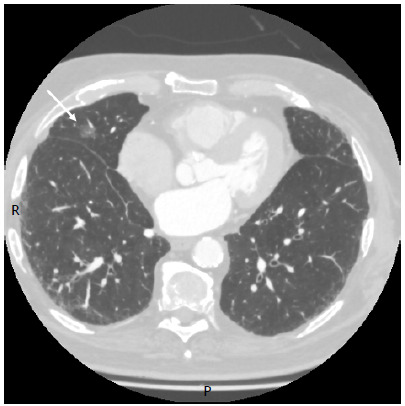
Ground-glass nodule in the lateral segment of the middle lobe, measuring 1.4 cm.

**Figure 2:**
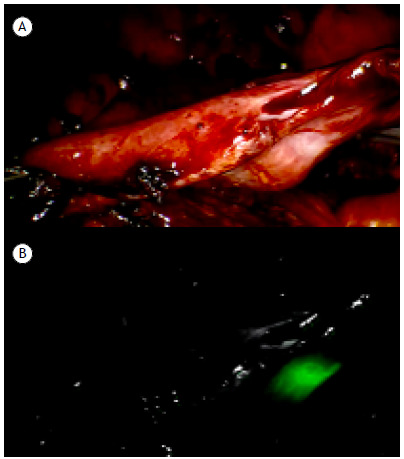
In A, posterior view of the lateral portion of the middle lung lobe, showing a nodule previously marked with indocyanine green before the use of a near-infrared fluorescence thoracoscope. In B, posterior view of the lateral portion of the middle lung lobe, showing the nodule as viewed with the near-infrared fluorescence thoracoscope.

Of the 18 patients analyzed in the present study, 14 (77.8%) underwent preoperative lesion localization in the hybrid operating room and 4 (22.2%) underwent preoperative lesion localization by CT (Figures 1 and 2). Thirteen patients (81.3%) underwent nodule marking using a 19-gauge coaxial needle (range, 18-22 gauge). Of the patients who experienced complications, 2 developed pneumothorax after nodule marking. This, however, did not change the surgical plan. Complications such as hemoptysis and hemothorax were not observed. 

In comparison with other liquid makers, ICG provides superior visualization (when compared with blue dyes), especially in patients with anthracosis.^4^ Dyes such as lipiodol may pose a risk of pulmonary or cerebral infarction because of their high viscosity.^4^ Furthermore, ICG is one of the least expensive dyes, making it easily available. 

According to the manufacturer, a potential risk of using TISSEEL in the lung is embolization; therefore, caution is advised when inserting the coaxial needle, in order to avoid blood vessels. Nevertheless, metal fiducial markers are more commonly associated with complications, including pneumothorax.^3^

The association of ICG with a hemostatic agent with tissue adhesive properties has solved the problem of overflow in our center, although more robust studies are warranted to confirm such benefits. Furthermore, this technique can be used with fiducial markers other than ICG. 

In conclusion, the use of ICG in association with a surgical hemostatic agent with tissue adhesive properties for RATS is valuable because it can increase the ability to locate small pulmonary nodules intraoperatively, thus reducing the rates of conversion to thoracotomy, without the inconvenience of positioning a C-arm between the robotic arms. 
